# Contextual Adaptation of a Complex Intervention for the Management of Cancer Pain in Oncology Outpatient Services: A Case Study Example of Applying the ADAPT Guidelines

**DOI:** 10.1002/pon.70132

**Published:** 2025-03-22

**Authors:** Olivia C. Robinson, Suzanne H. Richards, Emily Shoesmith, Simon Pini, Marie Fallon, Matthew R. Mulvey

**Affiliations:** ^1^ Faculty of Medicine and Health School of Medicine Leeds Institute of Health Sciences University of Leeds Leeds UK; ^2^ Department of Health Sciences University of York York UK; ^3^ Cancer Research UK Edinburgh Centre MRC Institute of Genetics & Molecular Medicine University of Edinburgh Edinburgh UK

**Keywords:** adaptation, cancer, complex intervention, outpatient services, pain management

## Abstract

**Objectives:**

Standardising pain assessment in oncology outpatient services (OOS) leads to improvements in patients' pain and quality of life. The Edinburgh Pain Assessment Tool (EPAT) is a standardised cancer pain management tool that has been implemented on inpatient oncology wards (the original setting). Routine use of EPAT reduced post‐surgical pain in cancer patients (the original scenario) and led to more appropriate analgesic prescribing. We describe here a case study of adapting the EPAT intervention for use in tertiary OOS in the United Kingdom (UK) National Health Services (NHS), using the ADAPT guidelines.

**Methods:**

The adaptation process followed Moore et al.'s ADAPT guidance: Step 1: We assessed rationale for adapting EPAT by reviewing existing literature of pain management in OOS. Step 2: Semi‐structured interviews with 20‐healthcare professionals (HCPs) to understand current practice and how the intervention might fit the new context (OOS). Step 3: Identified the ‘core’ and ‘peripheral’ components of EPAT, undertook four co‐design workshops with 7‐HCPs to reconfigure EPAT to fit OOS (adapted version is referred to as EPAT+). Four HCPs trialled the EPAT+ intervention in practice to refine the intervention.

**Results:**

Combining qualitative data from interviews with feedback from the co‐design workshops and preliminary testing the prototype intervention highlighted several key adaptation goals for EPAT+. These included: (1) reduce length/time to complete EPAT+ due to time constraints in outpatient appointments, (2) the importance of pain re‐assessment and using EPAT to facilitate patients to self‐monitor their pain at home, and (3) the creation of new peripheral components to support communication with primary care providers.

**Conclusions:**

Using a theoretical driven conceptual guidance provided important learning on how to adapt an existing cancer pain management tool to a new setting (OOS). The result is a novel complex theory‐ and evidence‐based intervention that will be formally tested in a cluster randomised pilot trial.

**Abbreviations**
EPAT: Edinburgh Pain Assessment Tool
HCPs: healthcare professionals
OOS: oncology outpatient services

## 1 Introduction

In healthcare settings, implementing interventions that have worked elsewhere can save human and financial resources [1, 2]. Adaptation is often more efficient than developing bespoke complex interventions for each new clinical context. Efficacy and effectiveness of an intervention is dependent upon implementation fidelity, which itself is dependent on the context in which the intervention is operating [2]. Successful implementation of existing interventions requires adaptation of the intervention components and systematic of these components within the context of the new clinical setting. Intervention adaption for a new setting can be ineffective when contextual differences between the old and new setting are not recognised [1, 2]. Thus, it is crucial that core intervention components or functions are identified and adapted appropriately to fit the new context. Engagement with stakeholders at every stage of the adaptation processes is essential to ensure relevance and fit [3, 4].The adaptation process should be documented to monitor fidelity of the intervention and make it easier to describe the adaptations and their impact if they are useful and effective [5, 6].

Existing research indicates that intervention effects do not always directly transfer to new contexts; therefore, guidelines have been developed to support adaptation [7]. Moore et al.'s [7] ADAPT guidance for adapting a complex intervention recommends: (1) the formation of an adaptation team (including experts with experience in the intervention and those with knowledge of the new context); (2) assessing the rationale for the intervention and considering intervention‐context fit; (3) planning for and undertaking the adaptation; (4) planning for and undertaking the evaluation; and (5) implementing and maintaining the intervention at scale [7].

This paper presents a case study example outlining how we used Moore et al.'s [7] ADAPT guidelines to adapt The Edinburgh Pain Assessment Tool (EPAT), a complex intervention for managing post‐surgical cancer pain. EPAT was developed for use on inpatient wards; the current paper outlines how this intervention was adapted to fit Oncology Outpatient Services (OOSs). This includes recognising the methodological and conceptual challenges involved in adapting the intervention for both a new setting (i.e., inpatient ward/outpatient clinic) and new clinical scenario (i.e., post‐surgical pain/chronic cancer‐related pain). Each stage of the research was informed by evidence and theory of the process of complex intervention adaption [7, 8, 9].

## 2 Methods

**FIGURE 1:**
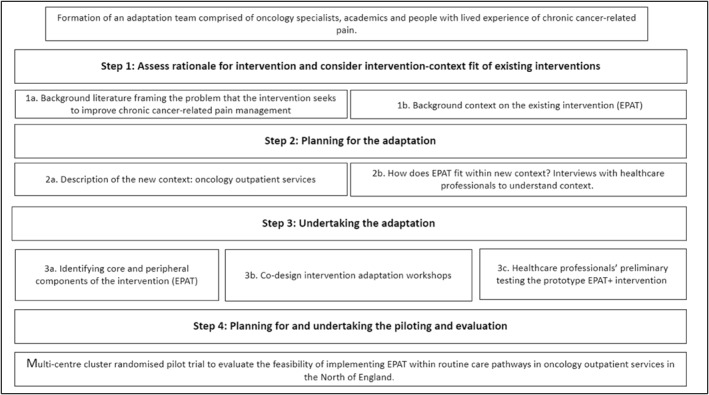
Adaptation process of EPAT following Moore et al.'s ADAPT guidance.

This paper follows the Moore et al.'s [7] ADAPT guidance with the following sections involving a systematic approach [4]. We used this guidance in conjunction with a combination of qualitative methods. We reviewed existing literature (step 1) and conducted semi‐structured interviews (step 2) and co‐design workshops with healthcare professionals (HCPs) and expert panel meetings with clinicians, academics, and patients to inform the adaptation process (step 3). An outline of the EPAT adaptation process using Moore et al.'s [7] ADAPT guidance is shown in Figure 1.

### 2.1 Formation of an Adaptation Team

An adaptation team was formed and supported by three expert panels:

- A panel of experts by patient experience comprised of three patients with experience of living with and managing chronic cancer‐related pain at home and two caring for relatives living with chronic cancer‐related pain.
- A panel of experts by clinical experience comprised of two oncology specialists (one consultant, one clinical nurse specialist) and three palliative care consultants. Clinical experts had direct experience of managing chronic cancer pain in OOS.
- A panel of experts by academic experience comprised of two complex intervention methodologist (authors S.H.R. and M.F.) and one qualitative methodologist (author S.P.). M.F. is the author of the original EPAT [10].

The panel of experts by experience were recruited through patient and public involvement groups through participating NHS trusts, and through existing patient and public involvement networks at the University of Leeds. The panel of experts by clinical experience were co‐applicants on the grant application, and the clinical experts were also embedded within the three NHS trusts that were recruited to participate in the study. The panel of experts by academic experience were co‐applicants on the grant application and embedded within methodology expert teams at the University of Leeds.

The three expert panels met with authors OR and MM 9 times between March 2022 and October 2023 to support them with the initial concepts for complex intervention adaption (Step 1, Figure 1), as well as providing contextual insights during the adaptation process (Step 2 and Step 3, Figure 1).

#### 2.1.1 Step 1: Assessing the Rationale for the Intervention and Considering Intervention‐Context of Outpatient Settings

##### 2.1.1.1 1a. Background Literature Framing the Problem That the Intervention Seeks to Improve

Despite numerous national and international guidelines on cancer pain management [11, 12], it remains a significant unmet clinical challenge. In the UK, one out of three people with cancer will experience moderate to severe cancer pain [13, 14]. In spite of support given to cancer patients at outpatient clinics, uncontrolled cancer pain is the most common reason for cancer patients to contact GP out‐of‐hours services [15]. However, when effective, systematic, cancer pain management strategies are integrated within cancer care pathways, these enable patients to access: (i) tailored drug treatments [10], (ii) support for self‐management [16] and (iii) referral for specialist help [17].

Guidance from the Faculty of Pain Medicine provides a framework for provision of pain service of adults with cancer [18]. This framework requires all oncology physicians to be able to screen and assess pain in a cancer patient at each clinical contact, provide a diagnosis of pain disorder, and provide analgesic management, supported self‐management and onward referral. However, there is no standardised, integrated process for managing cancer pain in secondary care OOS in the UK National Health Service (NHS) [19]. EPAT is the first systematic, integrated, cancer pain assessment and management intervention that meets the requirements of the Faculty of Pain Medicine cancer pain framework; that is EPAT includes components on pain screening, assessment and management, including onward referral [17].

Standardising pain control, with interventions like EPAT, is associated with earlier access to analgesia, advice for managing pain medicines, and receiving timely access to supportive and palliative care services [10, 20]. However, there has been little qualitative literature that provide detailed contextual description of usual/standard cancer‐pain management processes in OOS [19]. Therefore, we were unable to understand the extent to which the EPAT needed to be adapted to fit the new context based on existing published literature alone, and further qualitative data was required (see step 2a and 2b).

##### 2.1.1.2 1b. Background Context on the Existing Intervention (EPAT)

EPAT was designed to prompt clinicians to routinely and systematically assess and manage cancer‐related pain in a hospital ward setting [10]. EPAT comprises of the following core components: (1) a 0–10 pain intensity numerical rating scale (NRS) embedded within vital signs chart, (2) a detailed mechanism‐based pain assessment procedure, evaluating location, severity and type/mechanism of pain, (3) linked cancer‐pain specific treatment algorithms. These core intervention components, guided clinical decision‐making and treatment management plans.

Tested in a randomised cluster‐controlled trial, Fallon, Walker et al. [10] found EPAT significantly reduced cancer pain in patients on oncology wards and led to more appropriate analgesic prescribing, without higher doses. A key contextual factor for EPAT was implementation of EPAT within ward policy for post‐surgical signs and symptoms monitoring and management. This had two important consequences: firstly, the pain intensity screening NRS was embedded within the post‐surgical vital signs chart, which prompted regular (hourly) assessment and re‐assessment of pain to determine both efficacy and adverse effects of analgesic treatment [10]. The inclusion of EPAT in the patient's bedside chart encouraged ward staff to use EPAT every time the patients' vital signs were recorded. Subsequently, consultants had regular access to EPAT results when assessing patients (during ward round), facilitating consistent monitoring, and reviewing of pain. Secondly, a member of clinical staff was assigned the role of ‘EPAT champion’, who trained all clinical staff on the use of the intervention and maintained daily use of EAPT for all clinical staff.

The experts by experience panel helped identify key limitations in implementing EPAT in an outpatient setting without adaptation. For example:

- The vital signs chart was designed for inpatient care, including post‐surgical symptoms irrelevant to outpatients and patients living at home.
- The requirement for hourly assessment and re‐assessment did not align with the appointment‐based workflow of outpatient care.
- The original step‐two assessment, which focussed solely on pain location and severity, did not allow healthcare professionals to document additional relevant information.
- Step two also lacked an assessment of the underlying cause of pain, as it was originally designed for acute post‐surgical pain management.
- The treatment review chart was structured for inpatient settings, relying on daily multidisciplinary team meetings, which are not standard in outpatient care.

To successfully implement EPAT in an outpatient setting, it was clear that adaptations were necessary to align the intervention components with the new clinical environment.

#### 2.1.2 Step 2: Planning for the Adaptation

##### 2.1.2.1 2a. Description of the New Context

Outpatient care in the UK NHS is defined as clinical care that does not require patients to stay overnight for monitoring or supervision. Patients have several contact points with outpatient services throughout the process, during which components of an intervention for cancer pain management might be delivered. For example, prior to an outpatient appointment, patients receive information about their appointment, via letter, email, or text. Upon arrival at the oncology outpatient clinic, patient's check‐in with a receptionist or clinic clerk and wait until they are called. During this wait, patients are often seen by an HCP who may perform routine assessments (e.g., completing a symptom screening checklist).

During the appointment, patients will usually see a doctor or specialist nurse to discuss their symptoms, medical history, test results, and/or ongoing care plan. The doctor or specialist nurse can undertake necessary examinations and incorporate symptom management plans within their overall plan for ongoing care of the patient. Patients will then usually receive a consultation summary that outlines what was discussed and the plan for ongoing care. These stages are a relatively standardised and common structure to delivering healthcare via OOSs.

##### 2.1.2.2 2b. Interviews Undertaken With HCPs to Understand Context: ‘How Could EPAT Fit Within New Context?’

Qualitative interviews were conducted to understand existing clinical practice and service structure for cancer pain management in OOS at the level of the patient, clinician, service, and system. An understanding of these structural components enabled the mapping of individual intervention components onto the clinical care pathway.

###### 2.1.2.2.1 Participants

Healthcare professionals (HCPs) were recruited from oncology outpatient services across three National Health Service (NHS) trusts in Northern England. To be eligible, HCPs needed a minimum of 6 months' experience in managing cancer pain within an oncology outpatient setting. Purposive sampling was employed to ensure diversity in participants' roles, including oncologists and clinical nurse specialists (CNS). The sample also included staff from different grades (consultants and registrars) and a variety of outpatient subspecialties (lung, breast, and bowel).

A consultant is the most senior doctor in a hospital department who have final responsibility for patient care, make key clinical decisions and supervise junior doctors, including registrars. A registrar is a doctor who is in advanced speciality training who provide direct patient care and make clinical decision under the supervision of a consultant. This approach ensured the inclusion of a broad range of perspectives on cancer pain assessment, support, and management across different disease trajectories.

###### 2.1.2.2.2 Recruitment Processes

Eligible healthcare professionals (HCPs) were identified and recruited through co‐applicant HCPs embedded within clinical teams, who distributed study information packs (including an information sheet and consent form) via email to their entire teams. The information packs contained the research team's contact details (O.C.R./M.R.M.), and interested participants were invited to reach out for further details. Upon contact, the research team provided an overview of the study, addressed any questions, and scheduled an interview at a convenient time. Sample size was determined based on previous qualitative studies conducted in oncology OS [21, 22].

###### 2.1.2.2.3 Procedures

Interviews were conducted via telephone or video call, based on participant preference. At the start of each interview, O.C.R. obtained verbal consent, which was audio‐recorded and stored separately from the main interview recording.

###### 2.1.2.2.4 Data Analysis

Data analysis followed Braun and Clarke's thematic analysis approach. Interviews transcribed verbatim by O.C.R. and L.A. The analysis employed an inductive–deductive process, drawing insights directly from participant interviews. Preliminary analyses informed adjustments to the topic guide as patterns in the dataset were identified. O.C.R., M.R.M., and S.P. conducted initial coding and theme development. Themes and subthemes were then discussed in data analysis meetings with the research team and experts by experience group, reviewing supporting codes and quotes to explore their meaning and significance.

#### 2.1.3 Step 3: Undertaking the Adaptation

##### 2.1.3.1 3a. Identifying Core and Peripheral Components of the Intervention (EPAT)

Adaptation of existing complex interventions requires the identification and description of each individual intervention component, including the process of integration into routine clinical practice [8, 23]. Each component is then categorised as the ‘core’ or ‘peripheral’. *Core* components are the main evidence‐based ‘active’ components that define how the intervention works (sometimes referred to as the ‘mechanism of action’) to bring about an intended change in outcome(s). Generally, core components should not be modified during the adaptation process. *Peripheral* components are components that can be adapted to enable the intervention to be successfully integrated within the new context [8, 23].

Using the original EPAT [10] intervention, the research team reviewed and classified each intervention component as a ‘core’ or ‘peripheral’ component. These were mapped against the understanding of the new context (OOSs) using existing literature and interview data from HCPs [19]. Examples of core intervention components included: Pain scoring, understanding location and severity of pain, identifying type of pain (i.e., neuropathic, bone), re‐assessment of pain and effect of analgesia. Examples of peripheral intervention components included: vital signs chart, nausea weight and bowels chart, treatment algorithms, and the intervention champion role.

The aim of this process was to identify potential matches and mismatches between the intervention components and service structure to create a plan for the subsequent need for contextual adaptation. Once the core and peripheral components were identified, the research team discussed whether there were any new components required to facilitate implementation of the adapted intervention within the new clinical context. Version one of the prototype interventions was created, and comprised core intervention components, unchanged peripheral components, adapted peripheral components and newly developed materials.

##### 2.1.3.2 3b. Co‐Design Intervention (EPAT) Adaptation Workshops

To distinguish from the original EPAT, the adapted version is referred to as EPAT+.

Co‐design EPAT+ adaptation workshops were conducted with HCPs to explore the appropriateness of the intervention in the new context (OOS) and the feasibility of the intervention.

###### 2.1.3.2.1 Participants

HCPs were recruited from the same three NHS trusts in Northern England from step 5.2. Participants were eligible to take part if: (1) they were an HCP employed at one of the following OOS: lung, breast, GI, bowel, bone metastasis, haematology, prostate or head and neck; (2) had more than 6‐month experience working in OOS; and (3) had previously taken part in step 5.2 interviews.

###### 2.1.3.2.2 Recruitment Processes

Participants recruited to step 5.2 interviews [19] were invited to participate in a co‐design workshop. Potentially eligible participants were contacted via invitation email and provided written informed consent prior to participation. Participants had varied job roles (oncologist, clinical nurse specialist [CNS]), and different staff grades (consultant and registrar), and worked in a range of outpatient subspecialities.

###### 2.1.3.2.3 Procedures

The co‐design workshops took place remotely via video call in small groups (up to four people per group, including two researchers) and lasted approximately one‐hour. Prior to the workshops, an early‐prototype version of EPAT+ was shared with participants via email. During the workshops, participants were shown each individual component of the intervention on a PowerPoint presentation. Participants were asked to discuss and consolidate the core and peripheral components of EPAT+; discussions related to the duration, modality (paper/digital), and content of the EPAT+, including number of pain assessments, staff training, mode of delivery, potential outcomes for tailored pain management, and identification of user‐led solutions for implementing the EPAT+ within existing clinical care pathways. Workshops were audio‐recorded to check for accuracy and notes were taken during the workshops.

In a separate co‐design workshop, the expert patient panel were asked to review and comment on each intervention component and its implementation strategy. Feedback was elicited through discussion and documented by researcher fieldnotes.

###### 2.1.3.2.4 Data Analysis

Content analysis was conducted to analyse workshop data (i.e., fieldnotes, digital wall charts). The findings were translated and integrated into the EPAT+ materials, including the implementation strategy. This was an iterative adaptation process that occurred between each workshop and was supported by the three expert panels to ensure alignment with existing local clinical practice and clinical guidelines.

##### 2.1.3.3 3c. HCPs Preliminary Testing the Prototype EPAT+ Intervention

Oncology HCPs participating in the co‐design workshops undertook preliminary testing of the adapted EPAT+ in their clinical practice. They provided feedback regarding their experience of using EPAT+, including exploration of the individual components and any perceived difficulties with the tool. At this early stage of testing, feedback was not sought directly from patients, as EPAT+ is primarily a clinician facing intervention. Feedback sessions were conducted either in‐person or remotely for the research team to review and make appropriate amendments to the intervention.

## 3 Results

### 3.1 Results 2b. Planning for the Adaptation: Interviews Undertaken With HCPs to Understand Context: ‘How Could EPAT Fit Within New Context?’

Interviews were conducted with 20 HCPs from three NHS trusts, lasting between 30 and 45 min. The participant demographics and analysed interview data that has been described in step 5.2 are reported in detail elsewhere [19].

In summary, we found HCPs discussed their approaches to cancer pain management during consultations and the challenges of ensuring continuity of care beyond these encounters. Key findings included: (1) HCPs' clinical experience influenced their approach to pain assessment; (2) remote consultations limited the ability of experienced HCPs to conduct thorough pain evaluations; (3) responsibility for cancer pain management was often diffused among HCPs; (4) nurses played a key role in supporting patients with pain management; and (5) integrating multidisciplinary teams posed challenges to maintaining continuity of care for pain management.

### 3.2 Results 3a. Undertaking the Adaptation: Mapping Core and Peripheral Components of the Intervention (EPAT) Against Semi‐Structured Interviews With HCPs

A key finding from mapping the core and peripheral components against the interview data highlighted the challenges of the cyclical process of regular routine assessment and re‐assessment of pain (including impact of analgesic medications) in an outpatient setting. Assessment and monitoring of pain during outpatient appointments varied significantly (i.e., weekly, monthly or yearly), and time constraints during consultations made it difficult to appropriately discuss pain [19].

Feedback from HCPs highlighted which of the original EPAT components could be potentially categorised as core or peripheral in the new context (OOS). Additionally, HCPs highlighted the practical implications of adapting EPAT to the new context. For example, time constraints (i.e., 20‐min per outpatient consultations) restricted opportunity to explore pain management, and sometimes, was not considered a priority for discussion.

**FIGURE 2:**
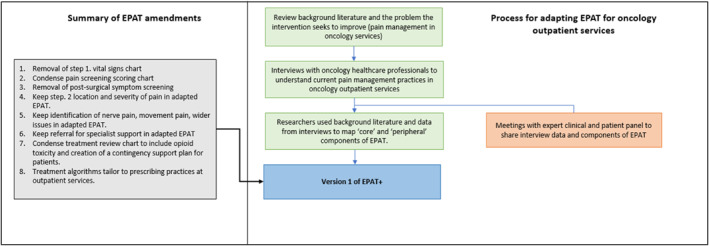
Flowchart of the intervention adaptation and data collection procedures for version one of the prototype interventions (EPAT+).

**FIGURE 3:**
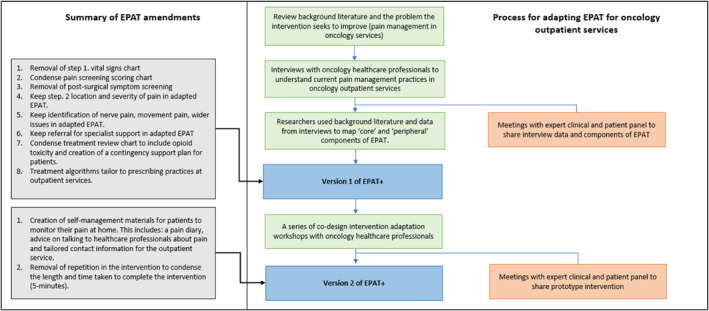
Flowchart of the intervention adaptation and data collection procedures for version two of the prototype intervention (EPAT+).

**FIGURE 4:**
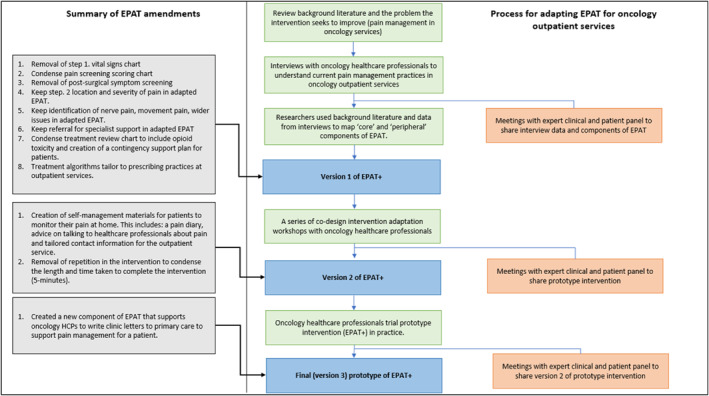
Flowchart of the intervention adaptation and data collection procedures for final (version 3) prototype intervention (EPAT+).

Table 1 describes the original EPAT components, whether they are considered core or peripheral, and outlines the interview findings and modifications made to each EPAT component to develop the first prototype of the adapted intervention (EPAT+). EPAT+ is summarised in Figure 2, and Figures 3 and 4 outline the developmental process of EPAT+ in line with step 1–3.

**TABLE 1:** Mapping existing components of the intervention (EPAT) onto the new clinical context (outpatient services).

Intervention (EPAT)	Core or peripheral	Qualitative data	Amendments to intervention
1. Step 1. vital signs chart	Peripheral	Outpatient settings do not usually monitor vital signs and facilitate re‐assessment of cancer pain	Remove vital signs chart
2. Pain screening scoring 0–10 numerical rating scale (NRS)	Core	Standard practice in outpatient settings is to carry out a pain screening using the NRS scale	Removal of extra input boxes as re‐assessment will not happen every 4‐h. Could be completed at every individual consultation or at telephone follow‐up
3. Post‐surgical symptom screening: Nausea, weight, and bowels chart	Peripheral	Weight, nausea, and bowel charts are not routinely recorded during outpatient consultations	Removal of weight and nausea score
4. Step 2. Location and severity of pain	Core	Asked at most consultations with a patient that is experiencing pain	Keep in adapted version of intervention
5. Identification of nerve pain	Core	Open‐ended conversations had with patients to understand type of pain	Keep in adapted version of intervention
6. Identification of movement pain	Core	Open‐ended conversations had with patients to understand type of pain	Keep in adapted version but rename to bone main
7. Identification of wider issues	Core	Open‐ended conversations had with patients to understand non‐pharmacological methods for pain management	Keep in adapted version of intervention
8. Referral for specialist support	Core	Open‐ended conversations with patients about specialist referral	Keep in adapted version of intervention
9. Treatment review chart (opioid toxicity, type of intervention, next date & time of pain assessment review)	Peripheral	Patient appointments varied monthly, weekly, yearly making it difficult to consistently review pain	Condense treatment review chart but incorporate new topics on adapted from to drive conversation with patients:
		Limited paperwork in consultations, making it difficult to keep a treatment review chart to regularly complete	Opioid toxicity Self‐management plan/contingency plan
		HCPs identified the importance of driving more discussions around opioid toxicity	
			3. Referral
10. Treatment algorithms	Peripheral	Less experienced HCPs acknowledged the benefits of having further support with prescribing complex pain relief	Update treatment algorithms to latest medical recommendations and tailored to the prescribing practices of hospitals

### 3.3 Results 3b and 3c. Undertaking the Adaptation: Adapting the Intervention (EPAT) Using Data From Co‐Design Workshops and Preliminary Testing of the Prototype (EPAT+) With HCPs

Seven HCPs participated in four co‐design workshops and four HCPs trialled the prototype intervention in OOSs. HCP demographics for both stages are presented in Table 2.

**TABLE 2:** HCP's demographics for co‐design workshops and preliminary testing of prototype intervention.

Healthcare professionals' characteristics (*n* = 11)	
Male	5
Female	6
Role	
Consultant	9
Clinical nurse specialist	1
Pharmacist	1
Cancer sub‐speciality	
Haematology	2
Lung	3
Gastrointestinal tract (GI)	3
Prostate	1
Breast	2

Amendments to the intervention following co‐design workshops and preliminary testing of the prototype intervention are presented in Table 3. Feedback from HCPs echoed a core component of the original intervention was prompting re‐assessment of pain [10]. We had feasibility conversations to understand the logistics of how the intervention would be delivered to patients. This prompted discussions for how re‐assessment of pain would be carried out when outpatient appointments were infrequent. It was deemed essential that EPAT+ was integrated into patient medical records so that clinicians can monitor changes of pain at future appointments.

**TABLE 3:** Amendments to intervention from co‐design workshops and service development feedback.

Intervention (EPAT)	Core or peripheral	Co‐design workshop and service development feedback	Amendments to intervention
1. Regular reassessment of pain to review effect of treatment intervention (i.e., analgesic medication)	Core	Feedback recommended value of including the EPAT forms on patient records to support re‐assessment of pain	Developed self‐management materials for patients which included a pain dairy to capture elements of re‐assessment of pain in‐between outpatient clinic appointments
		Useful to encourage patients to also monitor and self‐assess their pain at home so patients know when to contact the outpatient clinic for re‐assessment and review of treatment	Including a process to scan/file completed EPAT forms onto patient records so they can be reviewed by HCPs at the next outpatient appointment
2. Healthcare professional completing step 1 and 2 of the intervention	Peripheral	Feedback highlighted potential ways the EPAT form could be integrated into outpatient clinics	Step 1 of the intervention could be completed by a clinical support worker prior to the patient's consultation
		Experienced HCPs recommended utilising clinical support workers/healthcare assistants in the clinics to initiate the intervention	Step 2 needed to be completed by a healthcare professional with a prescribing qualification and thus was done during consultations
3. Length of EPAT	Peripheral	Feedback on first adapted protype of the intervention highlighted the need for the EPAT form to be condensed	This meant the intervention was condensed further. The research team removed any repetition and elements of the intervention that were not considered relevant by HCPs to the new context (i.e., treatment review chart)
		HCPs suggested it was repetitive and needed re‐shaping to include the core elements	
4. Frequency of delivery of EPAT	Peripheral	Feedback highlighted patients would not always return for consistent outpatient appointments but often are referred to primary care	Include additional section in adapted version called ‘clinic letter’ with points to consider when writing clinic letter

Secondly, we found that EPAT+ would benefit from new ‘self‐management’ component that aided patients to report pain earlier, aligning with previous research [24]. We developed new EPAT+ intervention materials that supported patients to self‐monitor their pain at home. This included how to describe pain to HCPs, a pain diary to identify pain changes and information on next steps (i.e., contact information for out‐of‐hours service, contacting clinic nurse) (Supporting Information S2—*managing cancer pain at home*).

Between the third and fourth co‐design workshop, feedback and input was received from the expert clinical panel. This facilitated the interpretation and integration of feedback from the first three workshops into an updated version of the prototype intervention (Version 2). Following the fourth co‐design workshop, EPAT+ (version 2) was presented to the expert patient panel for further feedback (Figure 3). The expert clinical and academic panels provided detailed descriptions of the clinical and managerial structure of OOS, including how EPAT+ could be integrated within existing standard‐operating‐procedures and operationalised within routine clinical practice these service structures.

The expert patient panel shared their experiences of communicating with HCPs about living with and managing cancer pain (i.e., ‘usual care’ from the patients' perspective), and their perceptions of the organisational structure of OOS. The expert patient panel also reviewed and provided feedback on the new ‘self‐management’ intervention materials from version 2 of the prototype intervention.

Feedback from using the prototype intervention (EPAT+ version 2) in practice led to new points for consideration. Outpatient settings often referred patients to primary care with an accompanying clinic letter describing discussions between oncology consultants and patients regarding ongoing treatment and management plans. Feedback from the preliminary testing of the prototype (EPAT+ version 2) showed EPAT was useful for informing the clinic letter to primary care about the patient's pain management (Supporting Information S1—*—Edinburgh pain assessment tool*).

### 3.4 Final Prototype Intervention (EPAT+)

The final prototype intervention (EPAT+) comprised of the following core components which remained the same with minimal adaptation from the original version (EPAT) to the adapted version: (1) a 0–10 pain intensity numerical rating scale (NRS); (2) a detailed pain assessment focussing on location, severity and mechanism; (3) treatment management algorithms; (4) guidance for continued management for cancer pain (e.g., onward referral to a pain specialist). A key mechanism of action of the original EPAT was systematic pain assessment and re‐assessment at each clinical contact. This was embedded within the hourly vital signs assessment and enabled regular assessment and review of patients' pain and response to treatment. We adapted this by adding a new intervention component, patient resources, which included self‐management support for monitoring pain at home between outpatient consultations and guidance on how to talk to their oncologist about their pain.

Figure 4 shows a flowchart of the intervention adaptation and data collection procedures outlined between step 1 (assessing the rationale for the intervention) to step 3c (HCPs preliminary testing the prototype intervention). Figure 4 also provides a summary of the changes made to EPAT described in step 6.1,6.2,6.3.

### 3.5 Step 4: Planning for and Undertaking the Piloting and Evaluation

An empirical feasibility pilot trial will be conducted. EPAT+ is designed to be implemented at the level of the OOS and used by HCPs with all patients attending the service. For service‐level intervention delivery cluster randomised trials are recommended [25, 26]. Cluster trials enable implementation at service‐level while minimising contamination between HCPs randomised to intervention or control arms of the trial.

## 4 Discussion

Our paper demonstrates a worked example of Moore et al.'s [7] ADAPT guidance to adapt a complex pain management intervention to fit a new context. While the ADAPT guidance is relatively new, using this guidance supported an evidence‐informed process to identify core and peripheral components and develop an adapted intervention that acknowledges barriers and enablers to supporting pain management in OOSs.

A strength of the ADAPT guidance [7] is the focus on understanding implementation in a new context. This echoes Medical Research Council (MRC) guidelines, and the importance of understanding mechanisms of change and/or resources required to support intervention reach and impact in real world implementation [9]. Research has shown stakeholder involvement in the co‐design of complex interventions can improve utility, usability, acceptability of the intervention and subsequent phases of intervention development (i.e., pilot phase) [3]. Integrating stakeholder experiences increases the likelihood of the tool being acceptable to the end user and to ensure the intervention can be integrated within routine clinical practice. We have proposed this case study so future researchers are able to see an operationalised example of using the ADAPT guidance [7]. There are few examples of how this guidance can be used to adapt existing interventions, therefore we proposed a strategy that combined a variety of qualitative methods.

### 4.1 Study Limitations

Defining core and peripheral intervention components has been challenged as being over‐simplistic and failing to recognise intervention complexity [2]. Disentangling a complex intervention into core and peripheral components is particularly problematic when viewed through a system thinking and realist evaluation lens [2]. To overcome this challenge, a pragmatic approach was adopted when adapting the core and peripheral components within the new context. This meant we not only described each component, but also its function in the original context. We employed participatory methods, to ensure that adaptation was informed and driven by the end users, who identified the core components of EPAT that were retained during its adaption to the new context.

Our approach to adapting a complex intervention could be considered resource intensive. There are also no established criteria for when to stop the intense adaptation process which makes it difficult to determine when it is appropriate to move on the pilot phase. This is a common challenge in intervention development [27]. However, by writing up the intervention adaptation process we will be able to make links between the intervention development process and the subsequent implementation fidelity of the intervention to learn from this.

## 5 Conclusions and Implications for Research/Practice

This paper contextualises Moore et al. [7]. adapting complex intervention guidelines and provides a case study example on how a cancer‐pain management intervention developed for oncology ward settings was adapted to fit a new outpatient context. Using these guidelines supported a theoretically and empirically informed process to define and develop an adapted intervention that acknowledges barriers to supporting optimal pain assessment and management in OOS. The result is a novel complex theory‐ and evidence‐based intervention that will be formally tested in a multi‐centre cluster randomised pilot trial.

## Author Contributions

M.R.M. is the chief investigator for this study, he conceived the project, led the design and writing of the study protocols, adapting EPAT and analysis, and draughting of this manuscript. O.C.R. led the writing of the study protocols, undertook completed data collection, adapting EPAT and analysis and wrote the first draft of the manuscript. All authors contributed to manuscript revision, read, and approved the submitted version.

## Ethics Statement

Ethical approval was obtained by University of Leeds, Faculty of Medicine Research Ethics Committee and Health Research Authority (21/HRA/5245). Approvals were also obtained at each NHS trust. Participants gave informed consent to participate in the study before taking part.

## Consent

The authors have nothing to report.

## Conflicts of Interest

The authors declare no conflicts of interest.

## Supporting information

Supporting Information S1

Supporting Information S2

## Data Availability

The authors have nothing to report.
