# Supplementary material for: Contextual Adaptation of a Complex Intervention for the Management of Cancer Pain in Oncology Outpatient Services: A Case Study Example of Applying the ADAPT Guidelines
Source: Psychooncology. 2025 Mar 22;34(3):e70132. doi: 10.1002/pon.70132 (PMC11929535; doi:10.1002/pon.70132)
Supplement: Supplementary file 1 — Supporting Information S1 [file PON-34-e70132-s001.pdf]

Date: ____________________

Stick Patient Label here:
Name… DOB…. NHS number

Edinburgh Pain Assessment Tool (EPAT©)

EPAT is designed to prompt conversations with patients about pain management.

Pain Score

Step 1: Pain Screening

"In the past 72 hours, how bad has your pain been at its worse?"

Worst Pain last 72 hours	(0-10) ____________________

O = No pain<br>1-2 = Mild pain	3 – 4 = Moderate pain (act)<br>Give analgesia<br>Use EPAT© Step 2	5-10 = Severe pain (act)<br>Give analgesia. Regular review until score is less than 3<br>Use EPAT© Step 2

Causes, Location & Severity

Step 2: Detailed Pain Assessment

Consider Underlying Causes of Pain…

Is the patient's pain primarily:

tumour-related pain ☐ treatment-related pain ☐ non-cancer related ☐

Consider Location and Severity…

On the body diagram, mark the sites where the patient feels pain.
If more than 1 pain, label pains A, B, C from worst to least painful and complete the box below

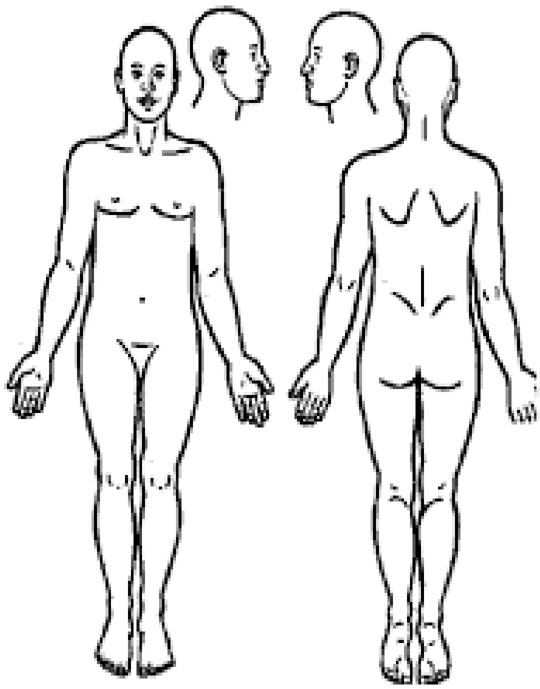

Pain Severity Score (0-10)	A Worst pain site	B	C Least pain site
Worst Pain in Last 24 hrs			
Least Pain in Last 24 hrs			

0 = No Pain 10 = Worst Pain Imaginable

Free text box to include pain descriptors:

Does the pain(s) disturb pain the patient's sleep? Yes ☐ No ☐

Nerve Pain

Consider Neuropathic Pain…

Is the patient's pain…? Shooting or Stabbing ☐ Tingling or Pricking ☐ Pins & Needles ☐ Hot or burning ☐ Allodynia ☐ Electric shock ☐

Do any areas of the patient's skin feel numb, strange or unpleasant to touch?
Yes ☐ No ☐ Detail: ______________________________

Consider neuropathic pain. Treat with adjuvants

Use 'Starting Gabapentin' or 'Starting Amitriptyline' EPAT algorithms

Bone Pain

Consider Movement Pain and Spontaneous Pain…

- Does moving or any other activity make your pain worse? Yes ☐ No☐
- Does your pain come on suddenly at rest? Yes ☐ No☐

Is the patient experiencing movement-related or spontaneous pain? **Consider bone pain**

Use WHO ladder – See 'Managing Cancer Induced Bone Pain' EPAT algorithm.

Give PRN analgesia before movement

Consider NSAID's / Palliative Radiotherapy / Bisphosphonates

Wider Issues

Consider Wider Issues…

- "What makes your pain better?" ____

Remember non-pharmacological interventions

Consider: Position change, Relaxation, Physiotherapy, Heat/Cold, TENS, Acupuncture

- ☹ Is there anything worrying or concerning you about your patient's pain? Discuss with patient.

Remember: ☹ anxiety/depression may co-exist with severe pain

Opioid Side effects

Consider opioid side effects…

- Is the patient experiencing any symptoms or side effects? Discuss with patient

Remember: there may be pain from constipation

Remember: Check for opioid side effects and toxicity

Side effects: ➢ Drowsiness ➢Constipation ➢Nausea

Toxicity: ➢ Confusion ➢Jerking/twitching ➢Hallucinations

Check-opioid dose may not suit

Remember… co-prescribing laxatives with opioids

Self-Management

Consider a contingency and self-management plan….

- What should the patient do if the pain persists? Discuss contingency plan with patient

Remember: Consider a contingency plan for patient if pain persists.

- Consider telephone follow-up
- Arrange a time/date for next appointment
- Provide patient with service telephone number and/or CNS/nurse contact number
- Provide suggestions for changes in medication.

Referral

Consider Referral

☎ Consider referral to your Specialist Team, i.e., palliative care or pain team, for patients who have:

- Persistent and/or severe pain
- Movement-related pain
- Pain unrelieved by initial management
- Opioid-induced drowsiness
- Require a rapidly increasing opioid dose

Clinic Letter

Consider including the below items in your clinic letter:

- Summary of pain symptoms
- Changes to medication
- Previous analgesics and how effective they were
- Actions for the GP
- Information given to the patient
