# Supplementary material for: Contextual Adaptation of a Complex Intervention for the Management of Cancer Pain in Oncology Outpatient Services: A Case Study Example of Applying the ADAPT Guidelines
Source: Psychooncology. 2025 Mar 22;34(3):e70132. doi: 10.1002/pon.70132 (PMC11929535; doi:10.1002/pon.70132)
Supplement: Supplementary file 2 — Supporting Information S2 [file PON-34-e70132-s002.pdf]

The Capture Study

Improving pain control for people living with cancer

A guide to managing cancer pain at home

Funded by Yorkshire Cancer Research

ctru University of Leeds

UNIVERSITY OF LEEDS

Contents

Introduction ... 3
Talking about pain to health professionals ... 4
Describing cancer pain ... 6
Monitoring your pain at home ... 9
Pain Diary Template ... 10
Contacts for advice and further information ... 12

Introduction

Having cancer does not always mean having pain, but if you do have pain there are things you can do to help monitor it at home so you can share this with your doctors and nurses so they can help you to control your pain.

Don't feel that you're being a nuisance or making a fuss by talking about your pain and how it affects you. Describing your pain can help your doctor or nurse understanding what it's like for you and plan the best ways to help you manage it. It might be helpful to have professionals answer your questions about your cancer and about the pain management methods you're using, or want to try.

Talking about pain to health professionals

Everybody is different when it comes to talking about pain. For some people it's natural to share experiences. Others might feel that they don't want to talk about being in pain. In this part we'll focus on talking to health professionals.

Being able to describe your pain to a doctor or nurse can help them to know what sort of pain you've got and what might help to relieve it. It might also be helpful to have professionals answer your questions about your cancer pain, what you are currently doing to manage it and what you might want to try in the future.

There is a wide range of health professionals you can talk to about your cancer pain, including:

- Oncologists and others involved in your cancer treatment
- Cancer Nurse
- GPs
- District nurses
- Community Pharmacists
- Pain specialists.

Sometimes it might not be clear who you should talk to, how you can get hold of them, or even what to say. If this happens, you could try talking to your hospital cancer team GP, or community nurse specialist (see page 12 for contact advice and further information. This will be to help you get urgent help if you need it)

Getting more help

Here are some tips about how to prepare what you want to say before you see the doctor, or nurse, or talk to them on the phone:

- Write down the most important points that you want to talk about on a piece of paper that you can take with you to appointments.
- Try to think of the most important thing you want to get across before you meet or talk to your doctor or nurse.
- Have a relative or friend with you when you meet your nurse or doctor. They can help you remember things the doctor or nurse says, and remind you of questions you may want to ask but forget.
- Make sure you understand what the doctor or nurse says to you about what can be done differently to try and relieve your pain. Ask for more explanation if there's anything you're not sure about.

It’s a good idea to summarise health professional’s answers – try saying something like ‘If I’ve got that right, what you’re saying is…’ This makes it clear how much you’ve understood, and can encourage the nurse or doctor to explain things more clearly if necessary.

- Ask for simple explanations. If you don’t understand, or if there is a lot to remember, it’s fine to ask the person to explain again and to go through everything point by point.

Some people are happy for you to record the discussion – on a mobile phone, for example – so that you can listen to it later.

You might want to ask the doctor or nurse to give you, or family members who are helping you, some written guidance about what you can do to relieve your pain.

Describing cancer pain

It's not always easy to describe pain, but there are some ways to describe pain that help your nurse or doctor to understand how it feels to you.

Sometimes people don't even use the word 'pain'. But describing the physical sensations in your body as clearly as you can, will help your doctors and nurses work out the best way of helping you manage them.

Here are some things you might think about to help you to describe your pain and discomfort – for example, you might try to describe where your pain is, how bad it is, what it feels like, and what makes it better or worse.

Describing where the pain is: you can use body diagrams to mark where your pain is. If you have more than one pain you may want to label them (e.g. A, B).

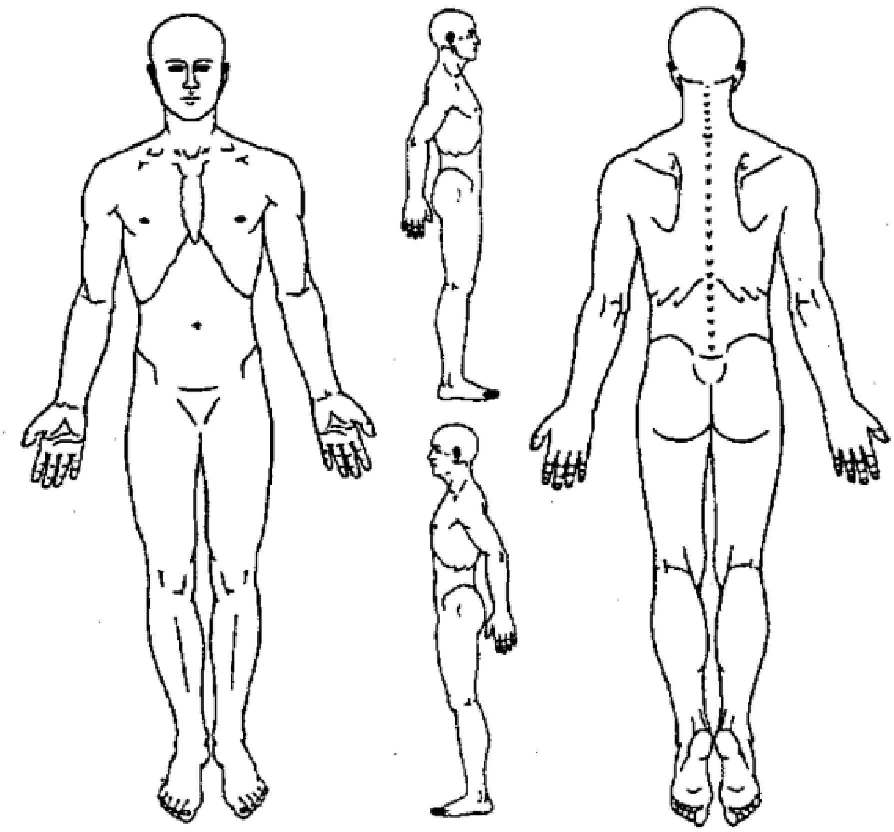

Describing how bad your pain is:

If you measured your pain on a scale of 0 to 10, how would rate it at this moment?

How bad was it at its worst in the last 24 hours? How bad was it at its least in the last 24 hours?

0	1	2	3	4	5	6	7	8	9	10
No Pain										Worst pain

At the end of this booklet is a pain diary you can use to track your pain throughout the day.

Describing what the pain is like:

For example, some people may describe their pain as a dull ache, tender, hot, burning, nagging, intense, sharp, shooting, stabbing, tingling, dull or throbbing to describe your pain.

Is your pain constantly there all the time, or does it come on and off suddenly like an electric shock?

In your own words:

Describing what makes the pain better or worse:

For example, is your pain worse when standing, sitting or lying down? Does moving or walking help to ease your pain, or does that make it worse?

Does a heat pad or ice pack help? Is your pain relieved by painkillers such as paracetamol? Do the painkillers stop the pain or just reduce it, and for how long?

Can you distract yourself with activities such as reading, listening to music, watching TV or playing video games?

In your own words:

Describe how your pain interferes with your day-to-day activities:

For example, does it stop you from moving around as usual? Can you bend or stretch if you need to? Does it stop you from sitting for very long – for example, can you sit long enough to eat a meal? Does the pain stop you from concentrating or affect your sleep? Does it stop you from walking for short or long distances.

In your words:

Describe how the pain makes you feel:

Does it make you feel anxious, frightened, depressed, irritated or hopeless? Do you have any positive feelings about pain? It can be hard to talk about these but it might help if you can tell some somebody about the way pain makes you feel?

In your own words:

Monitoring your pain at home

Monitoring your pain between consultations, can be helpful for your oncology doctor or nurse to see how you well your pain medicines are managing your pain. It can be helpful to keep a pain diary. These notes can help you and your health professional talk about how pain medicines are managing your pain and work out the best dose for you that fits into your daily routine.

There is no set way to record this information but it can be helpful to think about:

- Which medicines are controlling your pain and in what circumstances?
- Do you experience any side-effects, like constipation, nausea (feeling sick) or drowsiness?
- Do the medicines work a little or a lot? If you think one of the medicines takes the edge off the pain but is not controlling it, speak to your specialist nurse about this.
- What works well together? This could be heat pads, distraction and massage, in combination with the opioids.

When health professionals review someone's pain control they ask questions about the person's pain and how taking pain medicines fits into their daily routine. These details help health professionals' judge if a dose and strength of medicine is right for them. If the prescribed pain medicine is not controlling a person's pain, health professionals can change its dose and strength. At the end of the booklet there is an example of a pain diary that you can you use, if you wish.

Pain Diary Template

Date/ time	What were you doing? (i.e. driving)	Where was the pain?	What was the pain like?	Level of pain (0-10 rating)	Name and amount of medicine taken/ other pain control methods used	What worked and what didn't?	How long did the pain last?

Date/ time	What were you doing? (i.e. driving)	Where was the pain?	What was the pain like?	Level of pain (0-10 rating)	Name and amount of medicine taken/ other pain control methods used	What worked and what didn't?	How long did the pain last?

Contacts for advice and further information

Nurses and doctors are here to help when people want advice about managing their pain. The spaces below can be used to note down the contact numbers you might need. Your GP service, out of hours and oncology outpatient service can help with finding the right contact information.

People find it useful to keep these contact numbers or email addresses to hand, e.g. by their landline phone or stored in their mobile phone. Sometimes people need to contact a doctor or nurse outside of normal working hours. It can be helpful to discuss with a specialist nurse or doctor what 'out of hours' services are available.

GP

If you need medical advice or have an urgent query contact your GP. Try to contact your GP as early in the day as possible.

Name of GP or Surgery	
Contact number	
Email address	

Out of hours services

Sometimes people need advice about managing pain and their medicines outside of normal working hours. Your doctor or nurse will be able to tell you how to access your local out of hours services.

Out of hours service	
Contact number	
Email address	

Outpatient service

Some people need to contact their oncology outpatient service once they have left their appointment. Your outpatient service will be able to provide you with the best point of contact.

Outpatient service	
Contact number	
Email address	

Notes

This page has been left blank for you to write your own notes.

Back page

Notes

This page has been left blank for you to write your own notes.
